# Focusing Coherent Light through Volume Scattering Phantoms via Wavefront Shaping

**DOI:** 10.3390/s23208397

**Published:** 2023-10-11

**Authors:** Niklas Fritzsche, Felix Ott, Karsten Pink, Alwin Kienle

**Affiliations:** 1 Institut für Lasertechnologien in der Medizin und Meßtechnik an der Universität Ulm, D-89081 Ulm, Germany; felix.ott@ilm-ulm.de (F.O.); karsten.pink@ilm-ulm.de (K.P.); alwin.kienle@ilm-ulm.de (A.K.); 2 Faculty of Natural Sciences, Ulm University, D-89081 Ulm, Germany

**Keywords:** wavefront shaping, volume scattering, phantom, scattering coefficient, absorption coefficient, hybrid *P_N_* method

## Abstract

Manipulating the wavefront of coherent light incident on scattering media to enhance the imaging depth, sensitivity, and resolution is a common technique in biomedical applications. Local phase variations cause changes in the interference and can be used to create a focus inside or behind a scattering medium. We use wavefront shaping (WFS) to force constructive interference at an arbitrary location. The amount of light transmitted into a given region strongly depends on the scattering and absorption characteristics. These are described by their respective coefficients $\mu_{s}$ and $\mu_{a}$ and the scattering phase function. Controlling the scattering and absorption coefficients, we study the behavior of wavefront shaping and the achievable intensity enhancement behind volume scattering media with well-defined optical properties. The phantoms designed in this publication are made of epoxy resin. Into these epoxy matrices, specific amounts of scattering and absorbing particles, such as titanium dioxide pigments and molecular dyes, are mixed. The mixture obtained is filled into 3D-printed frames of various thicknesses. After a precise fabrication procedure, an integrating sphere-based setup characterizes the phantoms experimentally. It detects the total hemispherical transmission and reflection. Further theoretical characterization is performed with a newly developed hybrid $P_{N}$ method. This method senses the flux of light into a particular angular range at the lower boundary of a slab. The calculations are performed without suffering from ringing and fulfill the exact boundary conditions there. A decoupled two-path detection system allows for fast optimization as well as sensitive detection. The measurements yield results that agree well with the theoretically expected behavior.

## 1. Introduction

Imaging in complex, disordered media is often limited by scattering caused by local variations in the refractive index. The focus of light in turbid media degrades due to scattering and is attenuated by absorption. Light transport, and thus imaging results, is therefore blurred. Scattering in turbid media is a linear and deterministic process that can be used for inverse diffusion to force constructive interference at any point inside or behind the scattering medium [1,2]. This process is called wavefront shaping (WFS) and is based on the manipulation of the incident wavefront, where the phase and amplitude are varied. Knowledge of the optical properties and the structure of the medium under investigation allows direct access to its scattering behavior. Hence, sufficient information opens up the possibility of real-time WFS and therefore imaging inside and behind objects in the visible spectral region and beyond. For example, making the human body transparent to visible light is of great importance to many fields of research, development, and applied areas such as medicine [3].

This publication focuses on the behavior of WFS through volume scattering media with well-defined optical properties. In the literature, most experiments on WFS have investigated (quasi) two-dimensional samples, such as TiO${}_{2}$ or ZnO on coverslips [2,4,5,6,7] or PDMS compositions [8]. Other work has been conducted on intralipids [9] and chicken breasts [10]. To provide a template for any type of wavefront shaping experiment, the novelty presented in this paper is the complete process of designing, characterizing, and measuring well-defined volume scattering phantoms with predetermined optical properties. This level of control allows for a precise study of WFS and presents a method for the design of task-specific scattering and absorbing phantoms.

Section 2 presents the manufacturing process of these phantoms and their theoretical and experimental characterization. Section 3 describes the designed wavefront shaping setup, and Section 4 analyzes the measurements performed. Section 5 discusses the results obtained and lists some possible future applications.

## 2. Fabrication and Characterization of Phantoms with Well-Defined Optical Properties

### 2.1. Volume Scattering Phantoms

**Figure 1:**
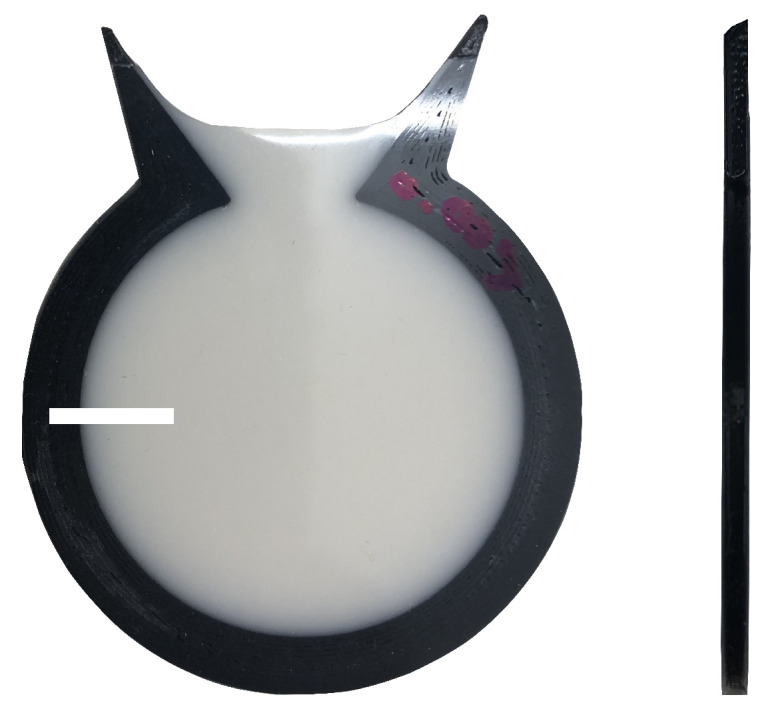
Front and side view with a scale bar of length 1 cm of an exemplary phantom made of epoxy resin filled in a 3D-printed flat open ring.

The principle underlying the fabrication process of the phantoms is the embedding of titanium dioxide (TiO${}_{2}$) particles as scatterers and molecular dyes as absorbers into a matrix of epoxy resin. This compound is then filled into 3D-printed frames of preset thicknesses. An exemplary phantom is shown in Figure 1.

In a process similar to [11], the volume scattering phantoms are fabricated following the below-mentioned steps. At first, the desired scattering and absorbing materials have to be chosen depending on the wavelength at which the experimental system is operating. The scatterers of choice for this work are TiO${}_{2}$ particles (Iolitec nanomaterials). These TiO${}_{2}$ particles provide strong scattering combined with minimal absorption for the operating wavelength of λ = 633 nm. The absorber used is an alcohol-soluble Nigrosin molecular dye (Sigma-Aldrich, St. Louis, MO, USA). Both components are easy to process with epoxy resin. The epoxy resin is a system of resin and hardener (SKresin72 and Epohard72, manufactured by SuK Hock GmbH, Regen, Germany) combined to provide the surrounding matrix for the scattering and absorbing particles.

Depending on the desired degree of scattering and absorption, a specific amount of TiO${}_{2}$ particles and molecular dye must be weighed to provide the required concentration. We perform several cycles of mixing the epoxy resin and the added components to avoid agglomerations in the compound. These would cause inhomogeneities in the optical properties of the phantoms. After mixing, the liquid compound is placed in an ultrasonic bath for several minutes, allowing the entrapped air to rise to the top of the sample. This is very important to ensure homogeneous optical properties and also breaks up agglomerations.

In the next step, several 3D-printed frames in the form of flat open rings with preset optical thicknesses are placed in between plates with surfaces as flat as possible. The selected form of the frames is needed to ensure that no air is trapped inside when filling these from the top. After filling, the epoxy resin has to harden for several days before the phantoms can be freed from the enclosing plates. Irregularities in the frames and the plate surfaces result in thickness variations across the sample surfaces. This had only a minor influence on our sample preparation but should generally always be taken into account.

For the experiments undertaken in the course of this publication, two sets of phantoms were created. The first set consists of phantoms with similar scattering coefficients, negligible absorption, and varying thicknesses. The second set features similar thicknesses and scattering, but the difference lies within the absorption coefficient.

### 2.2. Characterization of the Scattering and Absorption Coefficients

The characterization of the two sets of phantoms is performed using an integrating sphere setup and the methods described in [12,13]. A 3D-printed integrating sphere allows calibrated measurements of the total hemispherical transmission and the total hemispherical reflection. The optical properties are then calculated by a two-dimensional interpolation algorithm that compares the aforementioned measurement parameters with the corresponding parameters of a look-up table (LUT). This LUT has to be calculated beforehand by a Monte Carlo model taking several sample-specific parameters into account, such as an appropriate refractive index, the anisotropy factor *g* (the first moment of the scattering phase function, applying a Henyey–Greenstein function), and the sample thickness *d*. Of particular interest to the measurement results are the effective scattering coefficient $\mu_{s}^{{}^{\prime }}=\mu_{s}\left(1-g\right)$ and the absorption coefficient $\mu_{a}$. The two coefficients are determined with an accuracy for $\mu_{s}^{{}^{\prime }}$ of around 1% and 3% for $\mu_{a}$, given an appropriate optical thickness greater than one and a transmittance signal greater than 0.1% in the spectral range from 400 nm to 1500 nm [13]. These two sample-specific parameters combined with the respective thicknesses are used to classify the phantoms shown in the following Table 1. The parameters are listed with their respective uncertainties given by the standard deviation of three measurements at different sample positions.

### 2.3. Calculation of the Angular-Dependent Transmittance via the Hybrid $P_{N}$ Method

Following the fabrication process and experimental characterization with the integrating sphere, the obtained optical properties are used to calculate the angular transmittance from the phantoms. This is necessary to ensure a constant detected transmittance for each of them. Thus, one can directly compare the results obtained with wavefront shaping and analyze how well they match the theoretically expected behavior for the given parameters.

The authors would like to point out explicitly that only the aspects of the $P_{N}$ method relevant to this work and its application are presented below.

Fundamental work on the underlying $P_{N}$ method is based on the work of Case, Zweifel, and Pomraning [14]. The theory and Python code used in this work are based on a hybrid $P_{N}$ method, where the standard radiative transfer equation (RTE) is converted to a pure integral equation. The integral equation contains an integral kernel that fulfills the exact Fresnel boundary conditions instead of approximated formulas (or boundary conditions). Inserting the $P_{N}$ expansion into this equation then produces an alternative equation for the radiance based on the $P_{N}$ expansion moments. It can be evaluated directly at the boundary without suffering from ringing artifacts and is guaranteed to fulfill the exact boundary conditions there. This method, therefore, allows for the computation of the flux of light into a particular angular range at the lower boundary of a slab, which is very hard to achieve with the standard $P_{N}$ expression for the radiance.

The Python code used for the simulations takes into account $\mu_{s}^{{}^{\prime }}$, $\mu_{a}$, and *g*, assuming a Henyey–Greenstein scattering phase function. It also uses the refractive index of the sample and the refractive index of the surrounding medium as input. The former equals n=1.552, and the latter is given by the refractive index of air n=1. The simulation is verified using a Monte Carlo simulation with an incident pencil beam and a large detection area. This is valid because plane wave illumination and localized detection, as performed in the simulation, are equivalent to localized illumination and spatially integrated detection. Thus, there is no spatial dependence in either method, and the results of both methods can be compared and used for verification.

To be able to guarantee the applicability of the theory to the experiment, it is important that the k-vectors of the incident plane waves only hit the sample at comparatively small angles, which is ensured by the small numerical aperture (NA=0.4) of the first microscope objective. Therefore, the Ansatz of the plane waves incident on the slab geometry is in good agreement with the actual experimental environment.

**Figure 2:**
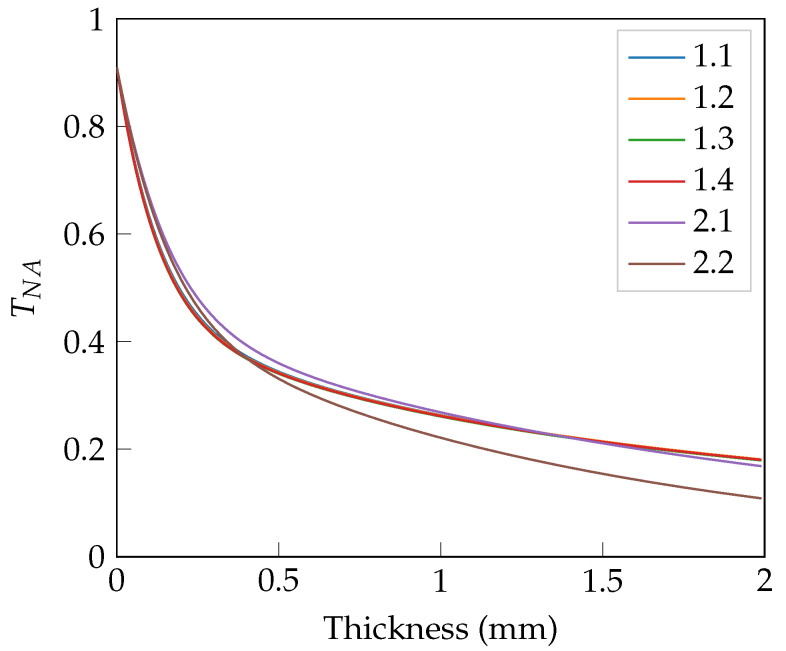
Partial angular transmittance $T_{NA}$ for the phantoms of set 1 and 2 plotted over thickness in mm, collected with a microscope objective of numerical aperture NA=0.8.

In the following, the angular transmittance from the lower sample boundary is calculated. By knowing the numerical aperture (NA) of the second microscope objective, which is compared in Section 3, the maximum angle transmitted into this aperture can be defined. Thus, the partial angular transmittance $T_{NA}$ can be calculated, which equals the angular transmittance integrated over the solid angle from zero degrees to the above-mentioned maximum angle. For the phantoms of set 1 and set 2, $T_{NA}$ is depicted in Figure 2. The laser output power is adjusted for each sample before experimental measurements using the partial angular transmittance. Therefore, a constant amount of light reaches the detection paths in the experimental setup despite the varying transmittance of the samples. Sample 1.2 combined with an a priori chosen laser output power is used as a reference.

**Figure 3:**
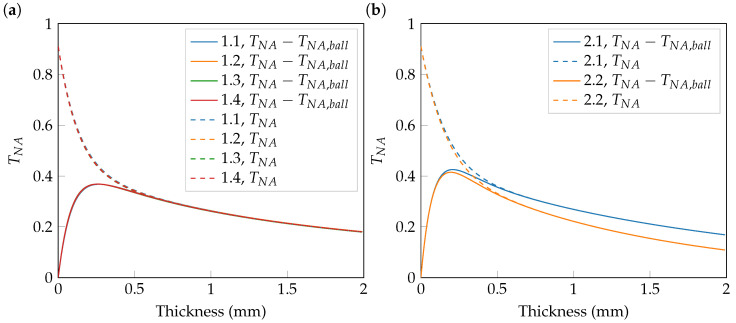
Partial angular transmittance for a microscope objective with NA=0.8 for set 1 (**a**) and set 2 (**b**) plotted over thickness in mm. Depicted are the transmittance into the NA without any ballistic contribution $T_{NA}-T_{NA,ball}$ and the partial angular transmittance $T_{NA}$.

As is depicted in Figure 2, the (optically) thinner the sample, the more light is transmitted in and near the forward direction, increasing the amount of light transmitted into the numerical aperture. In Figure 3a,b, the $T_{NA}$ for set 1 and set 2 is considered again in more detail. The two figures show the partial angular transmittance depicted over the sample thickness in mm. Together with the partial angular transmittance $T_{NA}$, the transmittance into the NA without any ballistic component given by $T_{NA}-T_{NA,ball}$ and is of particular interest. It is important to note that nonballistic light does not equal diffuse light. This is considered in more detail below. The ballistic component is given by a delta peak in the forward direction of $T_{NA}$ when illuminating the slab perpendicular to its surface normal.

Both figures show the expected dominance of the ballistic component $T_{NA,ball}$, especially for a thickness of 0 mm, since there is no scattering. The reason why the $T_{NA}$ does not start at $T_{NA}=1$ is that one has to take into account the loss of light caused by reflection and absorption, as well as the part that is not transmitted into the angular region of the detection NA. For small thicknesses, $T_{NA}-T_{NA,ball}$ rises to a maximum due to the conversion of ballistic light into diffuse light via scattering and a subsequent decay via absorption. The reflection increases as well for larger sample thicknesses. Wavefront shaping merely allows the manipulation of $T_{NA}-T_{NA,ball}$ because only this component undergoes phase randomization and does not preserve correlation, provided that a sufficient number of scattering events have taken place. The light, which experiences only a small number of scattering events (e.g., single or double scattering), cannot be treated as an utterly diffuse component and, thus, cannot be manipulated completely. Nevertheless, it is treated as a contribution to the diffuse component in this simulation’s framework. Sample 1.1 was intentionally chosen to be in the area where the ballistic component is present and the little scattered light is expected to be a nonsignificant contribution to the partial angular transmittance. Calculating the ballistic contribution $T_{NA,ball}$ of this sample to the partial angular transmittance, the hybrid $P_{N}$ method delivers a value of 2.02%.

In Figure 3b one can see the influence of absorption on $T_{NA}$, where a higher $\mu_{a}$ has a more distinct damping effect as expected.

## 3. Wavefront Shaping Setup

Coherent light passing through an opaque medium forms a speckle pattern due to interference caused by scattering. This process has long been known to be linear and deterministic [1,2] and can thus be manipulated to force constructive interference at an arbitrary location (e.g., behind the scattering medium, as is the case in this work). It can be performed in several ways [1], where the method of choice is phase-only manipulation, here accomplished with a Hamamatsu X15213 spatial light modulator (SLM).

**Figure 4:**
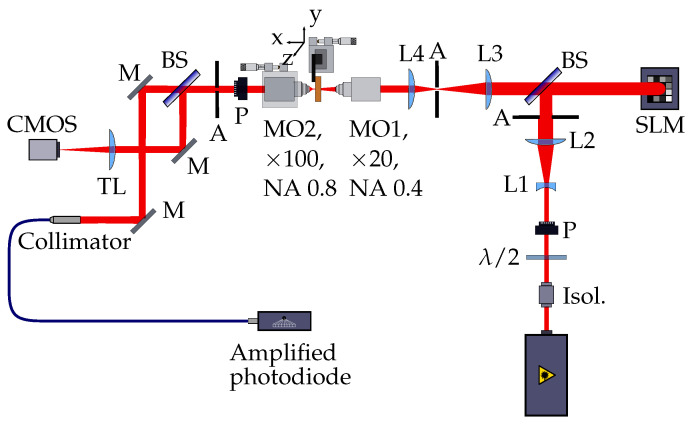
Wavefront shaping setup including a laser diode emitting at 633 nm, a spatial light modulator (SLM), microscope objectives (MO), a CMOS camera, and a gain-amplified photodiode. Isol., isolator; λ/2, half-wave plate; P, polarizer; L, lens; A, aperture; BS, beam splitter; M, mirror; TL, tube lens.

The system designed to undergo the following experiments is shown in Figure 4. A diode laser emits light at a wavelength of 633 nm that propagates through polarization optics at first. Adjusting the polarization is necessary because the SLM, located in the back focal plane of the first microscope objective, can only manipulate horizontally polarized light. The pixels of the SLM grouped into (*N*) square segments are arranged in a checkerboard pattern. Focused by the first microscope objective, the light transmits through the phantoms positioned so that the diameter of the illuminated area remains constant. Furthermore, the samples are always located in front of the focus concerning the optical axis. A consistently large illumination area allows a constant number of modes to be excited. However, the prerequisite is that the *N* segments remain linearly independent. This is tantamount to the light of neighboring segments not coupling into the same mode. More specifically, adjacent segments must have a sufficiently large k-vector spacing.

Divided into two paths, the scattered light collected with the second microscope objective then reaches the detection. A field aperture mounted behind the second objective matches the mean speckle size to the pupil of a multimode fiber (MMF) and ensures single speckle optimization. Before coupling into the MMF, the light is focused onto its end facette using a collimator. The signal transmitted through the fiber is transformed into a voltage signal and amplified, before being converted into a digital signal used as input for the optimization algorithm. In the second path, a tube lens images onto a CMOS camera within the spatial domain. This CMOS camera completes the feedback loop, measuring the peak-to-background ratio (PBR) calculated from the peak intensity of the optimized focal spot relative to the average background intensity for a series of random phase patterns. These patterns must have the same segment size as the optimized patterns to ensure consistent diffraction of the underlying grating structure. Locating the CMOS camera in a second detection path allows for independent measurements of the PBR while the optimization process is still running via the amplified photodiode. Thus, adequate settings for both paths can be chosen, which lowers the optimization time and increases the feedback signal.

Optimization and phase pattern calculation is performed with a stepwise sequential algorithm (SSA) [15] written in Python. This algorithm optimizes one segment at a time and adapts the resultant optimized phase pattern only for each iteration cycle. Therefore, it is much more sensitive to noise and has a lower contrast for each measurement compared to a differential equation genetic algorithm (DEA) [16] or a Hadamard encoding algorithm (HEA) [17]. However, since the noise in the detection part of the setup could be kept very low, the combination of an SSA and gain-amplified photodiode allows small changes in intensity to be detected very sensitively, which results in a slow convergence rate but high enhancement.

## 4. Results

With the characterization procedure described, the well-defined phantoms are now placed into the experimental setup to investigate the behavior of wavefront shaping through these volume scattering media. After adapting the laser output power, the results regarding the measured PBR are expected to follow the theoretical curve for the enhancement η according to Vellekoop et al. [2]. The formula is given by

(1) $$\eta =\frac{\pi }{4}\left(N-1\right)+1,$$

for phase-only manipulation, where *N* is the number of segments applied. The derivation of this formula assumes that the electric fields originating from each segment are uncorrelated and drawn from a random circular Gaussian distribution. Equation (1) results from a random walk with a large number of independent components. According to the central limit theorem, the statistic of *N* independent steps is asymptotically Gaussian [18] for N→∞.

**Figure 5:**
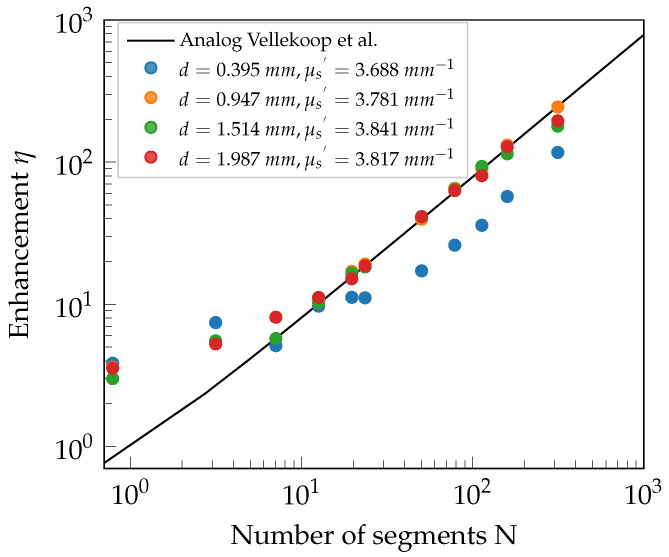
Measured intensity enhancement η as a function of the optimized number of segments *N* for the phantoms of set 1 with similar effective scattering coefficient $\mu_{s}^{{}^{\prime }}$, varying thickness *d*, and negligible absorption coefficient $\mu_{a}$. The experimental uncertainty is in the range of the symbol size [2].

**Figure 6:**
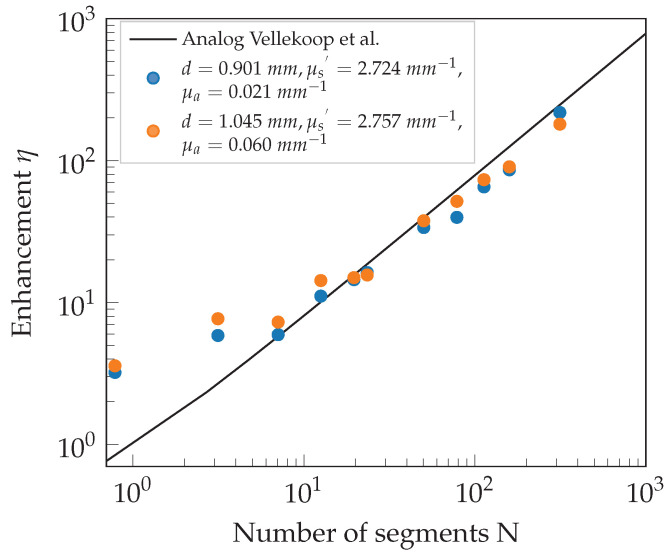
Measured intensity enhancement η as a function of the optimized number of segments *N* for the phantoms of set 2 with varying absorption coefficient $\mu_{a}$, similar effective scattering coefficients $\mu_{s}^{{}^{\prime }}$, and thickness *d*. The experimental uncertainty is in the range of the symbol size [2].

Furthermore, the residual amplitude modulation of the phase-only SLM results in an uncontrollable background of 5.6% in the intensity, which is measured similarly to [19,20]. The speckle decorrelation time is given with 2580 s, where a single measurement cycle takes about 0.025 s. Therefore, the system’s optimization speed is fast enough that the measurements are not affected by speckle decorrelation, so no corrections are added in Figure 5 and Figure 6 either. Averaging the measurements over several different sample positions reduces the experimental uncertainty to the order of the symbol size.

### 4.1. Solely Scattering Phantoms

The phantoms of set 1 mainly differ in their respective thicknesses, whereby their $\mu_{s}^{{}^{\prime }}$ is very similar in comparison with the uncertainty given by the standard deviation of 0.058 mm${}^{-1}$, and $\mu_{a}$ is negligible. Thus, they are treated as solely scattering phantoms, while only scattering effects are considered.

As long as the transport mean free path $\ell^{{}^{\prime }}=1/\mu_{s}^{{}^{\prime }}$ of each phantom is small compared to its thickness, the measured enhancement η is expected to follow Equation (1). The measurement confirms this assumption. The data points of phantom 1.1 in Figure 5 (blue dotted points) show the expected behavior of wavefront shaping when the optical thickness of the samples becomes too small to fully randomize the phase of an incident wavefront that undergoes scattering during transmission. The ballistically transmitted and little scattered light retains some phase correlation and does not undergo complete phase distortion. Hence, wavefront shaping is limited, which reduces the optimized focal intensity and increases the reference background. All in all, the enhancement becomes smaller for phantom 1.1, as is illustrated in the experimental results.

The measured intensity enhancements for the various phantoms agree well with the theoretically expected behavior from Equation (1) for both sets. Further experiments and theoretical considerations with more general boundary conditions are part of future work to characterize this effect in more detail.

The larger than expected enhancement values in both Figure 5 and Figure 6 for small *N* already appeared in measurements of Vellekoop et al. [2], and their exaggeration is due to the nature of the measurement process. An exemplary explanation will be given using the data point at N<1. The optimal phase pattern for this data point results in a constant phase offset across the area of the SLM used for optimization. Speckle vibrations during the phase sweep of the optimization process result in a random intensity exaggeration; thus, a higher-than-average intensity value is found for the singular optimized pattern. During the acquisition of the background, these random intensity exaggerations average out, and thus the enhancement results in a larger than theoretically expected value. However, this effect plays an increasingly minor role for a larger *N*. As shown in [2], the distance of the samples’ second boundary to the focus spot also has an influence that has to be considered.

The back aperture of MO1 limits the amount of light useable for WFS and defines the illuminated area on the SLM. When optimizing a single macropixel (N=1), a pixel array larger than this area is addressed. The overlap of both results in regions of manipulated but nonilluminated pixels, wherefore the effective *N* has to be adapted to N<1. Thus, the leftmost data points in Figure 5 and Figure 6 are located at N<1.

### 4.2. Influence of Absorption

Adding a substantial amount of absorption in addition to the already present scattering in set 2 allows us to observe scattering and absorption effects in combination. The additional absorption lowers the total transmission but should not affect the measured enhancement because both the focal intensity and the background are damped similarly. Confirmation is given by the measurements depicted in Figure 6. The data points mostly overlap for the phantoms with different $\mu_{a}$ and agree well with the theoretically expected behavior following Equation (1).

## 5. Conclusions

In summary, this work presented a complete and precise development process of well-defined volume scattering phantoms and their application for wavefront shaping. Starting from the desired optical properties $\mu_{s}^{{}^{\prime }}$ and $\mu_{a}$, along with the refractive index *n*, it has been shown that precise sample fabrication is feasible. A comprehensive method for experimental and theoretical characterization is presented using an integrating sphere and a newly developed hybrid $P_{N}$ method. Using the procedures listed here, precise control of the transmittance is possible. The relevant components are the angular transmittance, the partial angular transmittance in dependence on the freely selectable NA, and the ballistic contribution. In addition, the methodology presented provides a better access to and quantitative understanding of ballistic photons in the framework of volume scattering samples. Utilizing the hybrid $P_{N}$ method, an estimation is made of how diffuse the radiation is and, therefore, how achievable the theoretically expected enhancement is.

The expected experimental results and the behavior of WFS depending on the phantom properties are shown, where the measurements agree with the theoretically predicted values for optically thick samples. Hence, for any combination of strong scattering and a large thickness, the theory curve calculated by Vellekoop should be achievable with good agreement. This, however, is only valid under the condition that the transport optical thickness $\tau =\mu_{s}^{{}^{\prime }}d$ is sufficiently large and an adequate amount of light is transmitted. Besides the wavefront shaping experiments on phantoms with well-defined optical properties, WFS through three-dimensional samples of an a priori known microstructure will be the subject of future publications.

Following the steps presented, it is possible to create samples that mimic biological tissues for a particular wavelength, such as breast, muscle, skin, or fat tissue. Given the optical properties of such biological tissues, the hybrid $P_{N}$ method also allows for the calculation of the radiance inside those media. Therefore, the theoretically achievable intensity via WFS in an arbitrary location can be calculated, depending on the amount of available light.

In conclusion, phantoms with well-prescribed parameters not only allow for the possibility to mimic the properties of biological samples but also allow for a precise control of the transmittance. While ballistic and little scattered light in the course of wavefront shaping is, in the majority of cases, only perceptible in the form of an increase in the reference background and is not covered by the theory described in Section 4, the methodology presented offers the possibility of direct access and better quantitative understanding.

Building on this work and extending the design presented to multiple layers in both fabrication and characterization, even more complex samples consisting of different layers and varying optical properties are realizable. The phantoms can be used without many of the drawbacks of real biological tissues and provide a basis for realistic microscopy experiments. However, aspects remain that the phantoms we have designed do not cope with, for example, dynamic effects like blood flow, which also limit the optimization time.

Furthermore, the concept of broadband spectrally matched phantoms imitating a specific type of biological tissue can be challenging, especially for a multilayer system.

## Figures and Tables

**Table 1:** Thickness *d*, effective scattering coefficient $\mu_{s}^{{}^{\prime }}$, and absorption coefficient $\mu_{a}$ of the two sets of phantoms at λ = 633 nm. The values are shown with their respective standard deviations of three measurements at different sample positions.

Set.Sample	*d* (mm)	$\mu_{s}^{{}^{\prime }}$ (mm${}^{-1}$)	$\mu_{a}$ (mm${}^{-1}$)
1.1	0.395(9)	3.688(5)	0.0034(3)
1.2	0.947(4)	3.781(2)	0.0020(1)
1.3	1.514(9)	3.841(4)	0.0020(1)
1.4	1.987(3)	3.817(5)	0.0019(1)
2.1	0.901(8)	2.724(3)	0.0213(1)
2.2	1.045(3)	2.757(1)	0.0598(1)

## Data Availability

Data underlying the results presented in this paper are not publicly available at this time but may be obtained from the authors upon reasonable request.
